# Supplementary material for: Designed Rubredoxin miniature in a fully artificial electron chain triggered by visible light
Source: Nat Commun. 2023 Apr 25;14:2368. doi: 10.1038/s41467-023-37941-8 (PMC10130062; doi:10.1038/s41467-023-37941-8)
Supplement: Supplementary file 1 — Supplementary Information [file 41467_2023_37941_MOESM1_ESM.pdf]

Supplementary Information

Designed Rubredoxin miniature in a fully artificial electron chain triggered by visible light

Marco Chino[1], Luigi Franklin Di Costanzo[2], Linda Leone[1], Salvatore La Gatta[1], Antonino Famulari[3,4], Mario Chiesa[3], Angela Lombardi[1*] and Vincenzo Pavone[1*].

1. Department of Chemical Sciences, University of Naples Federico II. Via Cintia 21, 80126 Napoli, Italy.
2. Department of Agricultural Sciences, University of Naples Federico II. Via Università 100, 80055 - Portici (NA), Italy.
3. Department of Chemistry, University of Torino. Via Giuria 9, 10125 Torino, Italy.
4. Department of Condensed Matter Physics, University of Zaragoza, Calle Pedro Cerbuna 12, 50009 Zaragoza, Spain.

Correspondence to: alombard@unina.it; vipavone@unina.it

Table of Contents

Tables

Supplementary Table 1. Dihedral angles analysis of the 4-residue loops from MASTER search 2
Supplementary Table 2. Data collection and refinement statistics 3
Supplementary Table 3. Dihedral angles analysis of the ZnMETPsc1 crystal structure.... 4
Supplementary Table 4. H-bonds analysis of the ZnMETPsc1 crystal structure. 5

1. Supplementary Methods 6
1.1. Materials 6
1.2. Design of METPsc1 6
1.3. Solid phase peptide synthesis 13

2. Supplementary Results 15
2.1. Evaluation of the designed models and X-ray structure description 15
2.2. FeMETPsc1 redox cycling experiment time course 18
2.3. ZnMC6*a as photosensitizer in a photo-triggered electron cascade 18
2.4. Synthesis and purification 19

3. Supplementary References 21

Supplementary Table 1. Dihedral angles analysis of the 4-residue loops from MASTER search, showing that 14 and 3 fragments correspond to I’ and III’ beta turns, respectively.

	i+1		i+2		
**Structure Hit#**	**φ_2**	**ψ_2**	**φ_3**	**ψ_3**	**type**
wgap008	43.4	44	92.4	-0.9	I'
wgap009	-92.7	-141.4	-77.8	19.1	II'
wgap017	68.7	-118	-100.8	5.4	II'
wgap018	84.8	34.6	73.4	-6.2	I'
wgap020	49.2	36.4	87.3	-10.8	I'
wgap021	51.8	35.6	68.2	22.6	III'
wgap023	50.2	38.7	85.2	-16.7	I'
wgap025	-73.8	-12	-86.7	-25.9	I
wgap027	107.6	-25.8	-94.3	-49.3	nobeta
wgap034	63.1	36.6	65	12.3	III'
wgap036	110	-25.7	-128	-47.3	nobeta
wgap040	99.3	-10	-138.4	-33.1	nobeta
wgap042	-54.5	-34.7	-97.4	20	I
wgap046	-84.3	2.1	98	-20.1	V
wgap047	57.4	49.7	70.2	8.8	III'
wgap052	47.9	51.9	76.9	3	I'
wgap054	-83.4	-15	-80.3	-37.1	nobeta
wgap065	63.9	-121.7	-104.6	10.5	II'
wgap068	55.1	-133.3	-83.4	-4.8	II'
wgap071	49.4	33.9	79.5	6.6	I'
wgap072	59.7	30.1	80.2	5.6	I'
wgap081	56.5	50.7	91.6	-3.5	I'
wgap083	55.3	51.3	70.5	12.1	I'
wgap088	50.3	-145.3	-91.9	24.6	nobeta
wgap090	n.d.	n.d.	n.d.	n.d.	n.d.
wgap093	-78.3	-9.7	109.9	-4.3	I
wgap095	-111.7	-108	-88.6	1.4	nobeta
wgap098	54.2	41.1	85.9	-10.5	I'
wgap105	-101.2	73.3	62.4	12.2	nobeta
wgap109	-61.6	-24	-87.4	-46.2	III
wgap116	59.7	44.1	73.1	3.9	I'
wgap121	53.6	-139.7	-96.5	21.7	II'
wgap127	-108.6	-20	-130.1	110.2	nobeta
wgap131	110.5	-53.3	-88.5	-49.6	nobeta
wgap134	59.3	36.9	88.1	-41.9	I'
wgap140	-50	-31.2	-130.7	84	I
wgap145	53	52.8	75.9	-16.6	I'
wgap148	-56.5	-29.9	-89.1	-1.9	I
wgap152	79.4	69.3	69.1	3.5	I'

Supplementary Table 2. Data collection and refinement statistics.

**Data and refinement statistics**	**METPsc1-Zn(II)**
Wavelength (Å)	1.2400
Resolution, Å	31.2 - 1.34
Total/unique reflections	18,490/8,463
Completeness, % (overall/outer shell)	95.8/93.5
R_{merge} (overall/outer shell)	0.066/0.443
CC(1/2)	99.5
I/σ(I) (overall/outer shell)	7.55/1.48
Space group	$C222_1$
Unit cell dimensions (Å, Å, Å); Volume (Å^3)	37.663, 55.978, 19.128; 41,263
Refinement. No. of reflections, work/test	4,770/266
R/R_{free}	0.133/0.172
Protein atoms	383
Water molecules	23
R.m.s. deviations	
Bond lengths, Å	0.008
Bond angles, °	1.14
Dihedral angles, °	18.3
Ramachandran outliers, %	0
Ramachandran favored, %	100
Cβ deviations >0.25Å	0
RSCC/RSR (Zn ion)*	1.00/0.01
MolProbity all-atom clashscore**	2
Percentile score**	100
Mean B values, Å^2	
Protein atoms (whole chain)	17.6
Water molecules	26.6
Metal ion	12.9

* RSCC/RSR These values were obtained from the full wwPDB validation report compiled for the deposited coordinates through the wwPDB validation services. Real-space correlation coefficient (RSCC) and RSR are measures of the quality of fit between a part of an atomic model (in this case, zinc ion) and the data in real space (Jones et al., 1991). RSCC of 1.0 indicates a "perfect correlation".

** MolProbity clashscore These values were obtained from the full wwPDB validation report compiled for the deposited coordinates through the wwPDB validation services. The all-atom clashscore is defined as the number of clashes found per 1000 atoms (including hydrogen atoms) (Chen et al., 2010). There are no Ramachandran outliers to report (Percentile score).

Supplementary Table 3. Dihedral angles analysis of the ZnMETPsc1 crystal structure.

Residue	φ	ψ	Residue	φ	ψ
**Tyr1**	-152	158	**Tyr16**	-142	149
**Cys2**	-80	121	**Cys17**	-64	122
**Ser3**	-68	-24	**Thr18**	-76	-6
**Asp4**	-80	-36	**Asn19**	-90	-56
**Cys5**	-129	-10	**Cys20**	-107	-5
**Gly6**	91	-3	**Gly21**	78	10
**Ala7**	-61	150	**Ala22**	-61	146
**Asp8**	-64	139	**Ser23**	-63	151
**Aib9**	-50	-25	**Aib24**	-46	-38
**Ser10**	-62	-13	**Asp25**	-51	-27
**Gln11**	-99	17	**Arg26**	-81	-8
**Val12**	-100	134	**Ile27**	-76	138
**Arg13**	-152	145	**Arg28**	-132	162
**Gly14**	65	20			
**Gly15**	91	8			

Supplementary Table 4. H-bonds analysis of the ZnMETPsc1 crystal structure.

Residue	Atom	Residue	Atom	Distance
Ace0	O	Aib9	N	3.03
Tyr1	N	Arg28	O	2.77
Tyr1	O	Arg28	N	2.82
Tyr1	OH	Arg28	NE	3.58
Cys2	N	Ala7	O	2.82
Cys2	O	Gly6	N	2.76
Cys2	O	Cys5	N	3.52
Cys2	SG	Cys5	N	3.59
Cys2	SG	Asp4	N	3.38
Ser3	N	Arg26	O	2.94
Cys5	SG	Ala7	N	3.49
Asp8	N	Gln11	OE1	3.34
Asp8	O	Gln11	N	2.90
Asp8	OD2	Ser10	N	2.89
Aib9	O	Val12	N	3.29
Ser10	O	Arg13	NH1	2.96
Ser10	OG	Gln11	NE2	2.66
Gln11	O	Thr18	OG1	2.61
Gln11	O	Thr18	N	3.03
Arg13	N	Tyr16	O	2.99
Arg13	O	Tyr16	N	3.12
Gly15	O	Aib24	N	2.91
Cys17	N	Ala22	O	2.84
Cys17	O	Gly21	N	2.87
Cys17	SG	Asn19	N	3.42
Cys17	SG	Cys20	N	3.54
Cys20	SG	Ala22	N	3.46
Ser23	O	Arg26	N	2.92
Ser23	OG	Asp25	OD1	2.88
Aib24	O	Ile27	N	3.05
Asp25	OD1	Arg26	NH1	3.00
Asp25	OD1	Arg26	NH1	2.79
Asp25	OD2	Arg26	NH1	2.81

1. Supplementary Methods

1.1. Materials

All Fmoc (9-fluorenylmethoxycarbonyl) protected amino acids were purchased from Chempep. H-PAL ChemMatrix® resin and coupling reagents (HOBt, 1-Hydroxybenzotriazole, HATU, 1-[Bis(dimethylamino)methylene]-1H-1,2,3-triazolo[4,5-b]pyridinium-3-oxide hexafluorophosphate, and HCTU, 2-(6-Chloro-1-H-benzotriazole-1-yl)-1,1,3,3-tetramethylaminium hexafluorophosphate) were purchased from Sigma Aldrich and Novabiochem, respectively. All solvents, Acetonitrile, TFA (trifluoroacetic acid) and DIPEA (N,N-diisopropylethylamine) used in the synthesis and purification were anhydrous and HPLC grade, respectively, and were supplied by Romil. Piperidine and triisopropylsilane (TIS) were from Fluka. Mohr salt, 4-(2-Hydroxyethyl)piperazine-1-ethanesulfonic acid (HEPES), were purchased from Sigma Aldrich. Vydac supplied the columns C4-214TP1022 and C4-214TP5210. Data analysis was performed with OriginPro 9.0.0 software (Copyright 1991–2012 OriginLab Corporation, Northampton, MA, USA).

1.2. Design of METPsc1

Computational design of METPsc1 by miniaturization consisted of four different steps:

1) Generation of the backbone coordinates by symmetry.
2) Fragment search of the most designable closing loop.
3) Flexible-backbone sequence design: first round.
4) Flexible-backbone sequence design: second round.

Step 1: Initial backbone coordinates have been created using open source PyMOL[1] (version 1.8, Schrödinger, New York, NY, USA). The high potential mutant V44A from *Cp* Rd was used as initial template for the miniaturization procedure (pdb id: 1c09_A). First the PyMOL API was oriented around the tetrahedral iron center (selecting the metal and the four Sγ atoms). Subsequently, a copy of the segment V38-E50 was rotated counterclockwise around the Rd pseudo C_2 axis, as shown in the main text.

Step 2: We then focused in finding the best fragment linking the N- and C-termini of the two symmetric subunits. The MASTER software allows for the systematic search of non-contiguous tertiary motifs in a non-redundant protein database[2]. To perform this search, we selected two 3-residue fragments to build the query file (i.e., D47'-F49' and C39-L41,

at the N- and C-termini, respectively). PyMOL was used to save the excised query pdb, which was then converted into a pds file to perform the MASTER search against the database provided by the Grigoryan lab (v.2, https://grigoryanlab.org/master/). The following commands were used to perform these tasks:

```
createPDS --type query --pdb 1c09_symm_master1.pdb
master --query 1c09_symm_master1.pds --targetList ~/Public/MASTER_DB/list --rmsdCut
1.0 --gapLen '1-7' --structOut Search1 --outType wgap --bbRMSD --topN 1000 --
matchOut 1c09_search1.match --seqOut 1c09_search1.seq
```

where RMSD cut-off was calculated against backbone atoms of the query and set to a maximum of 1.0 Å, and gap length was comprised between 1 and 7 residues.

A total of 158 hits were retrieved, which were classified according to the number of residues in the bridging loop, and the RMSD from the query by Excel software (Copyright 1987-2021, Microsoft, Washington, US) (Supplementary Fig. 1). Data analysis showed that a I' beta-turn was the shortest yet designable choice to bridge the two symmetric subunits, with Gly residues at both i+1 and i+2 positions (Supplementary Fig. 2). To build the single-chain coordinates, the structure hit "wgap018.pdb" was selected (bbRMSD 0.69 Å), which features the desired conformation.

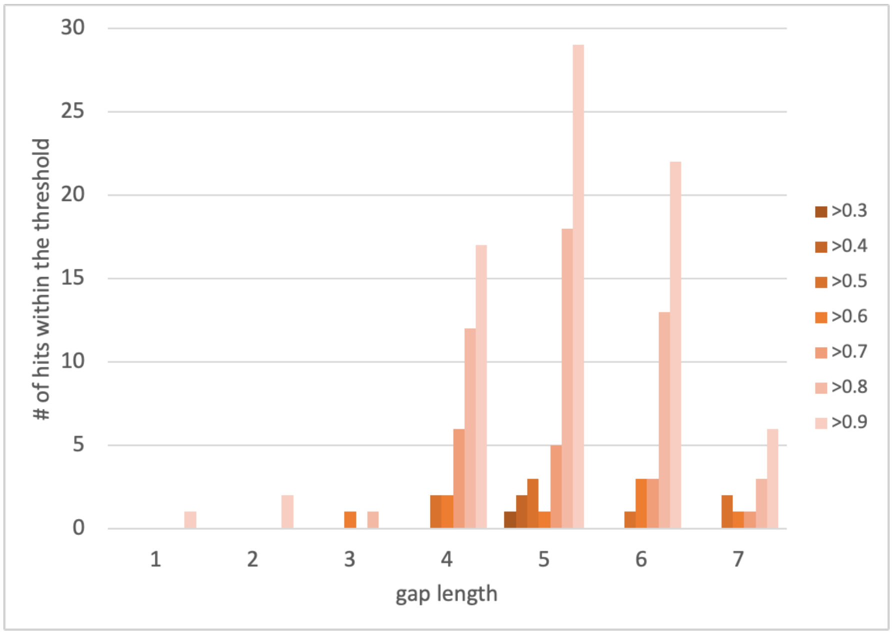

Supplementary Figure 1. Master search results. Total number of hits as a function of RMSD against the query structure.

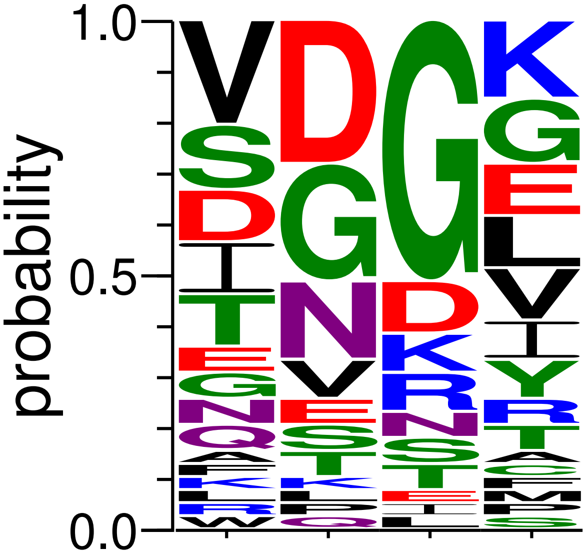

Figure 2. Sequence logo of the 4-residue fragments obtained from MASTER search.[3]

Step 3: ROSETTA software (rosettacommons.org, version 3.12, build 2020.08.61146) was used to perform a flexible design protocol under the Rosetta Scripts environment[4]. REF2015 was used as the scoring function[5]. A preliminary packing of the hydrophobic core (positions 9, 12, 24, 27) was intended to find the best residues fitting with the constraint of having Aib residues in positions 9 and 24. We found that the hydrophobic core was systematically asymmetric and mostly constituted by Phe, Leu, Val and Ile. For this reason, Val and Ile were chosen in position 12 and 27, respectively. The following command was given to run the flexible sequence design calculation, readapting a previously published protocol[6]:

```
rosetta_scripts.cxx11threadmpiserialization.linuxgccrelease
@q1_wgap18.generic.scripts.flags -s 1c09_symm_wgap18_AcNH2_ValIle.pdb -
multithreading:total_threads 4

q1_wgap18.generic.scripts.flags:
-nstruct 300
-out:path:pdb DESIGN_GENERIC/
-out:path:score DESIGN_GENERIC/
-out:file:renumber_pdb 1
-out:file:per_chain_renumbering 1
-out::file::pdb_comments
-run:preserve_header
-parser:protocol q1_wgap18.generic.xml
-chemical:exclude_patches NtermProteinFull NtermProteinFull_D2 NtermProteinFull_D
NtermProteinMethylated hbs_pre VirtualNterm a3b_hbs_pre CtermPeptoidFull
DimethylatedProteinCterm N_methylation CtermProteinFull

q1_wgap18.generic.xml:
<ROSETTASCRIPTS>
```

```
	<SCOREFXNS>
		<ScoreFunction name="hard" symmetric="0" weights="ref2015_cst">
			<Reweight scoretype="rg" weight="2"/>
			<Reweight scoretype="metalbinding_constraint"
weight="1.0"/>
		</ScoreFunction>
		<ScoreFunction name="hard_nocc" symmetric="0"
weights="ref2015_cst">
			<Reweight scoretype="rg" weight="2"/>
			<Reweight scoretype="coordinate_constraint" weight="0"/>
			<Reweight scoretype="metalbinding_constraint"
weight="0.0"/>
		</ScoreFunction>
		<ScoreFunction name="hard_cart_nocc" symmetric="0"
weights="ref2015_cst">
			<Reweight scoretype="rg" weight="2"/>
			<Reweight scoretype="coordinate_constraint" weight="0"/>
			<Reweight scoretype="cart_bonded" weight="0.5"/>
			<Reweight scoretype="pro_close" weight="0"/>
			<Reweight scoretype="metalbinding_constraint"
weight="0.0"/>
		</ScoreFunction>
		<ScoreFunction name="soft_nocc" symmetric="0"
weights="ref2015_soft">
			<Reweight scoretype="rg" weight="2"/>
			<Reweight scoretype="metalbinding_constraint"
weight="1.0"/>
			<Reweight scoretype="coordinate_constraint" weight="0"/>
		</ScoreFunction>
	</SCOREFXNS>
	<RESIDUE_SELECTORS>
		<Index name="cys" resnums="2,5,17,20"/>
	</RESIDUE_SELECTORS>
	<TASKOPERATIONS>
		<InitializeFromCommandline name="init"/>
		<IncludeCurrent name="current"/>
		<ReadResfile filename="resfile_generic_revised.txt" name="rrf"/>
		<ExtraRotamersGeneric ex1="1" ex2="1" extrachi_cutoff="0"
name="ex1_ex2"/>
		<RestrictToRepacking name="repackonly"/>
		<OperateOnResidueSubset name="cys_only">
			<Index resnums="2,5,17,20"/>
			<RestrictAbsentCanonicalAASRLT aas="C"/>
		</OperateOnResidueSubset>
		<OperateOnResidueSubset name="gly_only">
			<Not>
				<Index resnums="2,5,17,20"/>
			</Not>
			<RestrictAbsentCanonicalAASRLT aas="G"/>
		</OperateOnResidueSubset>
		<OperateOnResidueSubset name="prevent_Cys">
			<Index resnums="2,5,17,20"/>
			<PreventRepackingRLT/>
		</OperateOnResidueSubset>
		<OperateOnCertainResidues name="fixpolar">
			<ResidueHasProperty property="POLAR"/>
			<PreventRepackingRLT/>
		</OperateOnCertainResidues>
		<OperateOnCertainResidues name="fixcharged">
			<ResidueHasProperty property="CHARGED"/>
			<PreventRepackingRLT/>
		</OperateOnCertainResidues>
	</TASKOPERATIONS>
	<CONSTRAINT_GENERATORS/>
```

```
	<FILTERS>
		<PackStat confidence="0" name="ps" repeats="10" threshold="0.60"/>
	</FILTERS>
	<MOVERS>
		<ConstraintSetMover add_constraints="true"
cst_file="tetrahedral_metal.cst" name="ideal_metal"/>
		<SetupMetalsMover metals_detection_LJ_multiplier="1.0"
name="setup_metals"/>
		<SetupMetalsMover constraints_only="1"
metals_detection_LJ_multiplier="1.0" name="setup_metals_1"/>
		<FastRelax min_type="lbfgs_armijo_nonmonotone" name="relax_hard"
scorefxn="hard" task_operations="init,ex1_ex2,prevent_Cys">
			<MoveMap jump="0" name="r_hard">
				<Span bb="1" begin="1" chi="1" end="28"/>
			</MoveMap>
		</FastRelax>
		<Idealize coordinate_constraint_weight="0.1"
ignore_residues_in_csts="10A,11A,12A,13A,14A,15A,16A" name="ide"/>
		<PackRotamersMover name="softpack" scorefxn="soft_nocc"
task_operations="init,current,rrf,ex1_ex2"/>
		<PackRotamersMover name="hardpack" scorefxn="hard"
task_operations="init,rrf,ex1_ex2"/>
		<PackRotamersMover name="repack" scorefxn="hard"
task_operations="init,repackonly,prevent_Cys,ex1_ex2"/>
		<MinMover bb="0" bondangle="0" bondlength="0" cartesian="1" chi="1"
jump="0" max_iter="2000" name="sidechain_hard" omega="0" scorefxn="hard_cart_nocc"
tolerance="0.01" type="lbfgs_armijo_nonmonotone">
			<MoveMap jump="0" name="r_cart">
				<Span bb="0" begin="1" chi="1" end="28"/>
				<ResidueSelector bb="0" bondangle="0"
bondlength="0" chi="0" selector="cys"/>
			</MoveMap>
		</MinMover>
		<MinMover bb="1" bondangle="1" bondlength="1" cartesian="1" chi="0"
jump="1" max_iter="2000" name="mainchain_hard" omega="1" scorefxn="hard_cart_nocc"
tolerance="0.01" type="lbfgs_armijo_nonmonotone">
			<MoveMap jump="0" name="r_cart">
				<Span bb="1" begin="1" chi="0" end="28"/>
				<ResidueSelector bb="0" bondangle="0"
bondlength="0" chi="0" selector="cys"/>
			</MoveMap>
		</MinMover>
		<FastRelax bondangle="0" bondlength="0" cartesian="0"
min_type="lbfgs_armijo_nonmonotone" name="relax_hard_nocc" scorefxn="hard_nocc"
task_operations="init,ex1_ex2,prevent_Cys">
			<MoveMap jump="0" name="r_hard">
				<Span bb="1" begin="1" chi="1" end="29"/>
			</MoveMap>
		</FastRelax>
		<Backrub name="backrub" pivot_residues="1-28" require_mm_bend="1"/>
		<Sidechain name="sidechain"
task_operations="init,repackonly,fixpolar,fixcharged"/>
		<RandomMover movers="backrub,sidechain" name="rm"
weights="0.75,0.25"/>
		<GenericMonteCarlo mover_name="rm" name="backrub_mc" preapply="0"
scorefxn_name="soft_nocc" temperature="1.9" trials="200"/>
		<FilterReportAsPoseExtraScoresMover filter_name="ps" name="psr"
report_as="PSTAT"/>
	</MOVERS>
	<PROTOCOLS>
		<Add mover="setup_metals"/> #setting metal bonds and constraints
		<Add mover="softpack"/> #prepacking soft repulsion
		<Add mover="ide"/> #idealization of the beta-turn
		<Add mover="setup_metals_1"/> #re-setting metal constraints
```

```
                <Add mover="ideal_metal"/> #setting tetrahedral constraints
                <Add mover="relax_hard"/> #fastrelax protocol
                <Add mover_name="backrub_mc"/> #backrub protocol
                <Add mover="softpack"/> #packing with soft repulsion
                <Add mover="mainchain_hard"/> #minimize backbone
                <Add mover="sidechain_hard"/> #minimize side chains
                <Add mover="hardpack"/> #packing
                <Add mover="relax_hard_nocc"/> #fast relax without coordinate const
                <Add mover="hardpack"/> #packing
                <Add mover="psr"/> #packstat
        </PROTOCOLS>
        <OUTPUT scorefxn="hard_nocc"/>
</ROSETTASCRIPTS>
```

```
resfile_generic_revised.txt:
NOTAA HCM EX 1 EX 2
start
2   A   NATRO  #coordinating
5   A   NATRO  #coordinating
17  A   NATRO  #coordinating
20  A   NATRO  #coordinating
9   A   NATRO #AIB
24  A   NATRO #AIB
3   A   NOTAA KRLVIFCHM # prevent +charged and hydrophobic and cys-met-his
4   A   NOTAA KRLVIFCHM # prevent +charged and hydrophobic and cys-met-his
18  A   NOTAA KRLVIFCHM # prevent +charged and hydrophobic and cys-met-his
19  A   NOTAA KRLVIFCHM # prevent +charged and hydrophobic and cys-met-his
12  A   PIKAA V # hydrophobic core
27  A   PIKAA I # hydrophobic core
```

```
tetrahedral_metal.cst:
AtomPair SG 2A ZN 29A HARMONIC 2.3 0.1
AtomPair SG 5A ZN 29A HARMONIC 2.3 0.1
AtomPair SG 17A ZN 29A HARMONIC 2.3 0.1
AtomPair SG 20A ZN 29A HARMONIC 2.3 0.1
Angle SG 2A ZN 29A SG 5A CIRCULARHARMONIC 1.911 .2
Angle SG 2A ZN 29A SG 17A CIRCULARHARMONIC 1.911 .2
Angle SG 2A ZN 29A SG 20A CIRCULARHARMONIC 1.911 .2
Angle SG 5A ZN 29A SG 17A CIRCULARHARMONIC 1.911 .2
Angle SG 5A ZN 29A SG 20A CIRCULARHARMONIC 1.911 .2
Angle SG 17A ZN 29A SG 20A CIRCULARHARMONIC 1.911 .2
```

From this run, the following conclusions could be deduced (Supplementary Fig. 3): (i) the 2 corner residues of the newly-inserted I' beta-turn that links the two pseudo-symmetric halves of the model mostly corresponded to Gly (residues 14 and 15); (ii) N-terminal capping positions of the two 3_{10} helices were generally occupied by Ser, and Asp residues (positions 8 and 23), whose identities are also correlated to the *i+2* residues (positions 10 and 25, respectively); (iii) C-terminal capping positions of the two 3_{10} helices were generally occupied by Arg, forming the desired H-bond interactions (residues 13 and 28); (iv) Tyr residue, useful to concentration determination, was found at position 13, and to a lesser extent at positions 11 and 16; (v) the alpha-turn corner positions[7] *i+1* (residues 3

and 18), *i+2* (residues 4 and 19) were mainly occupied by Ser/ Thr, and Asp/Thr/Asn, respectively.

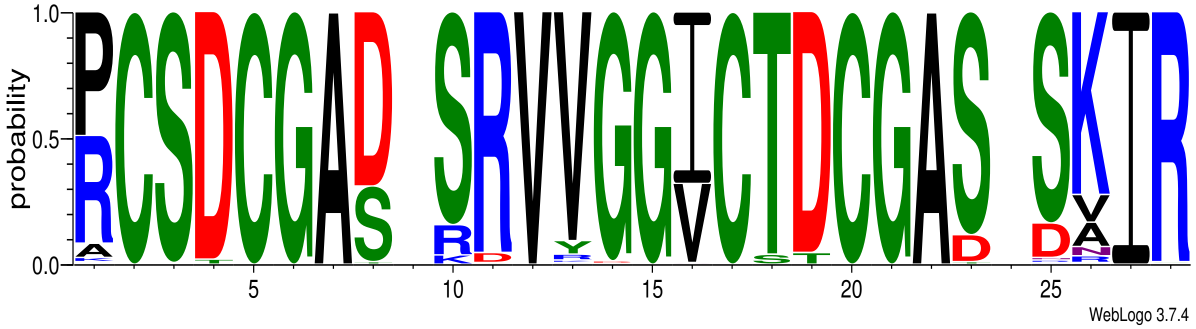

Supplementary Figure 3. Sequence logo of the first round of sequence design (empty positions are occupied by Aib residues).[3]

Step 4: A second design round has been performed to better explore H-bonding interactions around the coordinating Cys residues. This time a more permissive Monte Carlo conformation sampling, by means of the backrub protocol[8], was performed, by increasing temperature factor from 1.9 to 2.0. Design instructions were given in a new resfile. Only positions 1, 11 and 16 were left designable to look for best position for aromatic residue insertion, whereas other positions were kept fixed according to the results of the previous run. In choosing the latter residues, pseudo-symmetrization was used as a general principle. In particular: (i) alpha-turn corners were chosen to be “Ser3-Asp4” on the one side, and “Thr18-Asn19” on the other; (ii) 3_{10} helices were “Asp8-Aib9-Ser10” on the one side, and “Ser23-Aib24-Asp25”; (iii) C-term capping of 3_{10} helices was granted by Arg in positions 13 and 28; (iv) Arg was chosen in position 26 to make a salt bridge with Asp4 (as almost ubiquitously found in the pseudosymmetric position 11 from the previous run).

```
resfile_generic_revised_2ndRound.txt:
NOTAA HCM EX 1 EX 2
start
2   A   NATRO  #coordinating
5   A   NATRO  #coordinating
17  A   NATRO  #coordinating
20  A   NATRO  #coordinating
9   A   NATRO #AIB
24  A   NATRO #AIB
12  A   PIKAA V EX 1 EX 2 # hydrophobic core
27  A   PIKAA I EX 1 EX 2 # hydrophobic core
3   A   PIKAA S EX 1 EX 2 # alpha-turn corner i+1
4   A   PIKAA D EX 1 EX 2 # alpha-turn corner i+2
18  A   PIKAA T EX 1 EX 2 # alpha-turn corner i'+1
19  A   PIKAA N EX 1 EX 2 # alpha-turn corner i'+2
6   A   PIKAA G EX 1 EX 2 # beta bulge
```

```
7    A    PIKAA A EX 1 EX 2 # 2nd sphere
8    A    PIKAA D EX 1 EX 2 # 3_10 N-capping
10   A    PIKAA S EX 1 EX 2 # 3_10 end
21   A    PIKAA G EX 1 EX 2 # beta bulge
22   A    PIKAA A EX 1 EX 2 # 2nd sphere
23   A    PIKAA S EX 1 EX 2 # 3_10 N-capping
25   A    PIKAA D EX 1 EX 2 # 3_10 end
13   A    PIKAA R EX 1 EX 2 # 3_10 C-capping
28   A    PIKAA R EX 1 EX 2 # 3_10 C-capping
26   A    PIKAA R EX 1 EX 2 # salt-bridge
14   A    PIKAA G # type I' beta-turn
15   A    PIKAA G # type I' beta-turn
```

Sequence logo of the design result showed that position 16 was more amenable for Tyr residue than position 11 (Supplementary Fig. 4). Therefore, Tyr residues have been chosen in pseudosymmetric positions 1 and 16. Whereas, Gln was chosen in position 11.

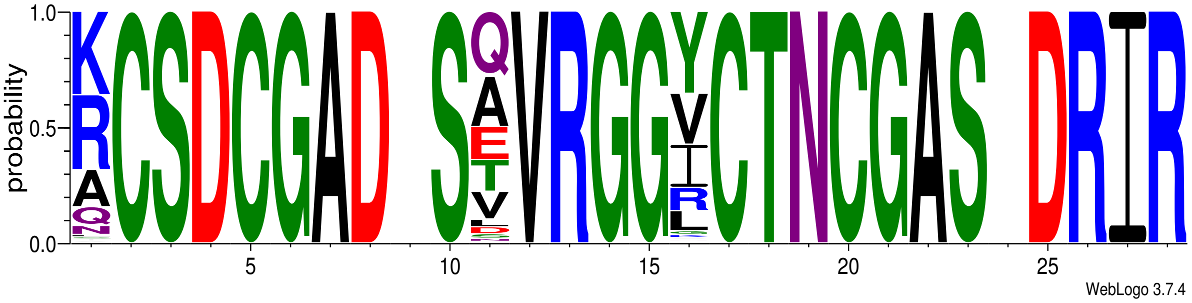

Supplementary Figure 4. Sequence logo of the second round of sequence design (empty positions are occupied by Aib residues).[3]

1.3. Solid phase peptide synthesis of METPsc Synthesis

METPsc1 peptide is made up of 28 residues with the following sequence:

Ac-Tyr-Cys-Ser-Asp-Cys-Gly-Ala-Asp-Aib-Ser-Gln-Val-Arg-Gly-Gly-Tyr-Cys-Thr-Asn-Cys-Gly-Ala-Ser-Aib-Asp-Arg-Ile-Arg-NH_2

The peptide was synthesized by automatic solid-phase synthesis using an ABI 433A peptide synthesizer (Applied Biosystem, Foster City, CA, USA) with Fmoc-protocols on a 0.1 mmol scale. The N- and C-termini were acetylated and amidated, respectively. The resin used was acid labile H-PAL ChemMatrix with a substitution of 0.20 mmol/g.

The following protected amino acids were used:

Fmoc-Arg(Pbf)-OH; Fmoc-Asp(OtBu)-OH; Fmoc-Aib-OH; Fmoc-Ser(tBu)-OH; Fmoc-Ala-OH; Fmoc-Gly-OH; Fmoc-Cys(Trt)-OH; Fmoc-Asn(Trt)-OH; Fmoc-Thr(tBu)-OH; Fmoc-Tyr(tBu)-OH; Fmoc-Val-OH; Fmoc-Gln(Trt)-OH; Fmoc-Ile-OH.

The synthetic procedure can be summarized as follows:

- **Deprotecion**: N-α Fmoc deprotection was accomplished with a solution of 20% *v/v* piperidine in NMP. After deprotection, the resin was washed with NMP to remove the piperidine.
- **Activation**: the carboxyl group of each Fmoc-amino acid was activated by direct addition in cartridge of 1 mmol HATU.
- **Coupling**: the pre-activated Fmoc-amino acid were coupled using a 0.5 M HOBt solution in DMF. In the coupling step, the activated Fmoc-amino acid reacts with the amino-terminal group of the growing peptide chain to form a peptide bond. Single coupling was conducted for each amino acid, except for the ones with particularly hindered lateral chains.
- **Capping**: this reaction was performed after each coupling step, using Ac_2O/HOBt/DIEA solution in NMP, to prevent the formation of deletion by-products.

Deprotection, coupling and capping steps were repeated with each subsequent amino acid, until the chain assembly was completed. The N-terminal amino group was acetylated with Ac_2O/HOBt/DIEA solution in NMP. When the synthesis was complete, the resin was washed with NMP, methanol and finally dried.

Cleavage from the resin and sidechain deprotection was achieved with a mixture of trifluoroacetic acid/H_2O/triisopropylsilane/ethanedithiol 9.4:0.25:0.25:0.1 (v/v/v/v). The reaction was carried out under moderate stirring for the first hour in an ice bath and then for the second hour at room temperature. The resin was then washed with TFA. After partial evaporation using a rotovapor, the crude peptide was precipitated, centrifugated and washed three times with diethyl ether, and finally dried.

2. Supplementary Results

2.1. Evaluation of the designed models and X-ray structure description

The final model was obtained by performing a final repacking/backrub/relax protocol[9], and selecting the structure with the lowest energy score. All the Rosetta designed models were compared to the Rosetta minimized X-ray structural model by using the same score function and weights (Supplementary Fig. 5). The designed models clustered to a lower energy minimum, suggesting that the conformational subspace has been sufficiently explored. Interestingly, Rosetta output models do not reach RMSD values below ~0.4 Å, which may be ascribed to some limitations in the metal binding scoring/constrains.

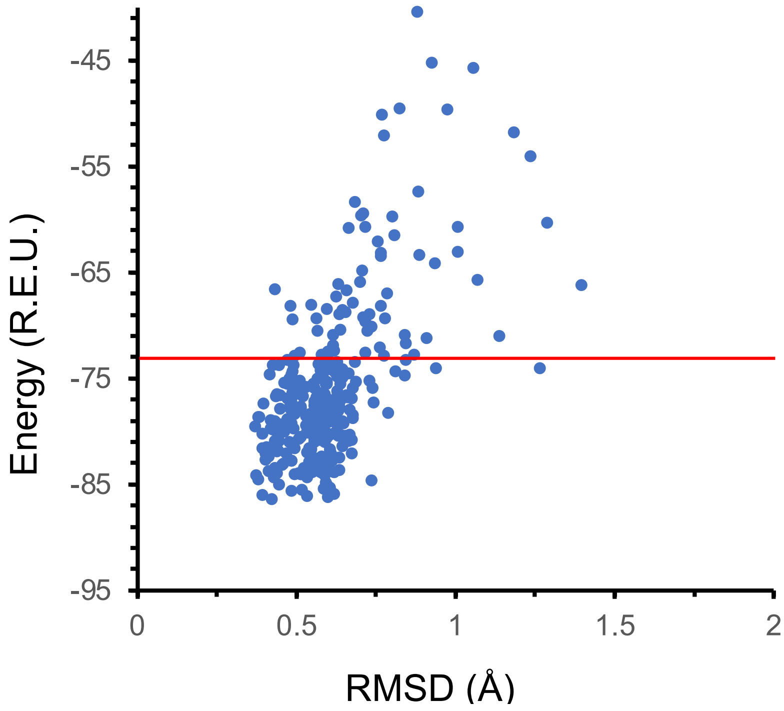

Supplementary Figure 5. Energy *vs* RMSD plot of the Rosetta relaxed ZnMETPsc1 models. Blue points correspond to designed models; red line defines the Rosetta score as calculated for the crystal structure.

The ZnMETPsc crystal structure is characterized by an unusual topology (Supplementary Fig. 6), unprecedented in the PDB, and a truncated cone shape (Supplementary Fig. 7).

In fully agreement with the design, the overall fold features two 3-stranded antiparallel β-sheets sandwiching against each other with a twist angle of ~110°. The two sheets are composed by three β-hairpin motifs[10] and a C-terminal strand: two are featuring the Iα_{RS} α-turn[11] followed by a β-bulge, also known as type AAAa π-turn[12] (between strands A-B and D-E), and one with the I' β-turn[13] (strands C-D). The C-terminal strand (Strand F) folds antiparallel with

respect to the first strand, bringing the N- and C-termini close together in H-bonding distance. The two 3_{10}-helices (green strands 1-2) lay on both sides of the β-sandwich enclosing the hydrophobic bottom in a substantially flat parallelogram formed by Cα atoms of Aib9, Val12, Aib24 and Ile27.

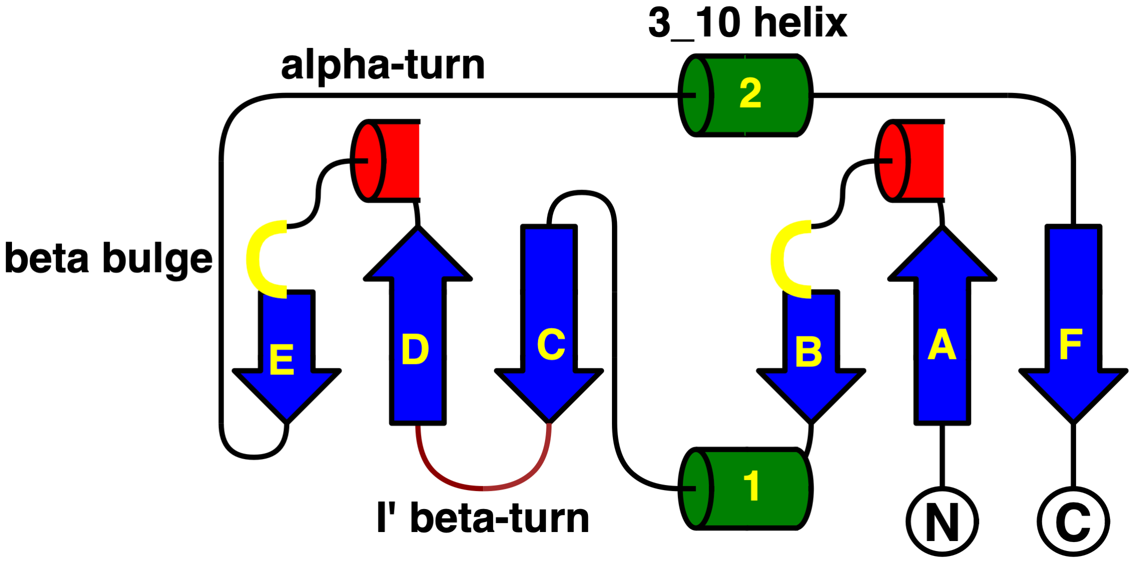

Supplementary Figure 6. Topology diagram of ZnMETPsc1 designed model. Blue arrows correspond to the six β-strands from A to F, where A starts at the N-terminus and F closes at the C-terminus; yellow curls correspond to Gly6/21 β-bulges; small red tubes correspond to α-turns; brown junction between strands C and D correspond to the β-turn; green tubes 1-2 correspond to the 3_{10}-helices.

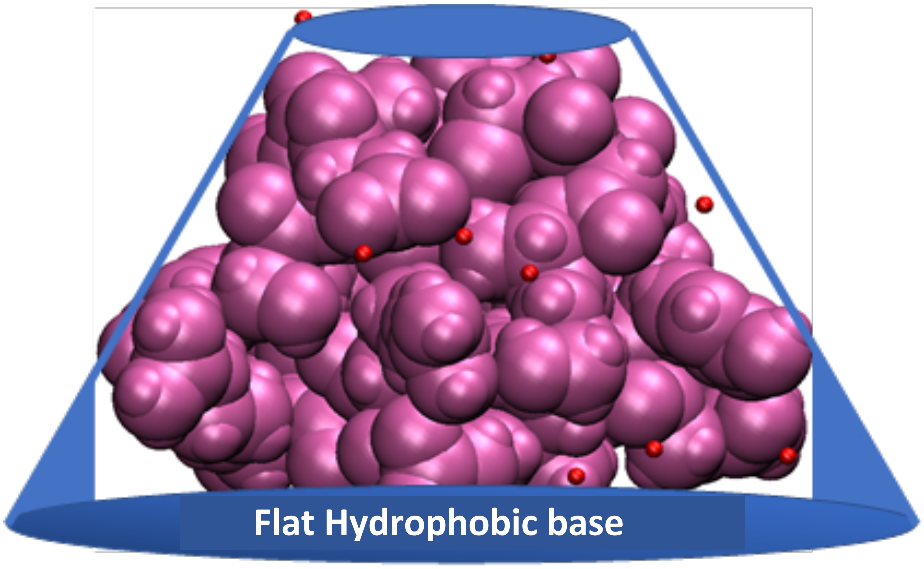

Supplementary Figure 7. Truncated cone shape of the folded METPsc1 molecule

2.2 FeMETPsc1 redox cycling experiment time course

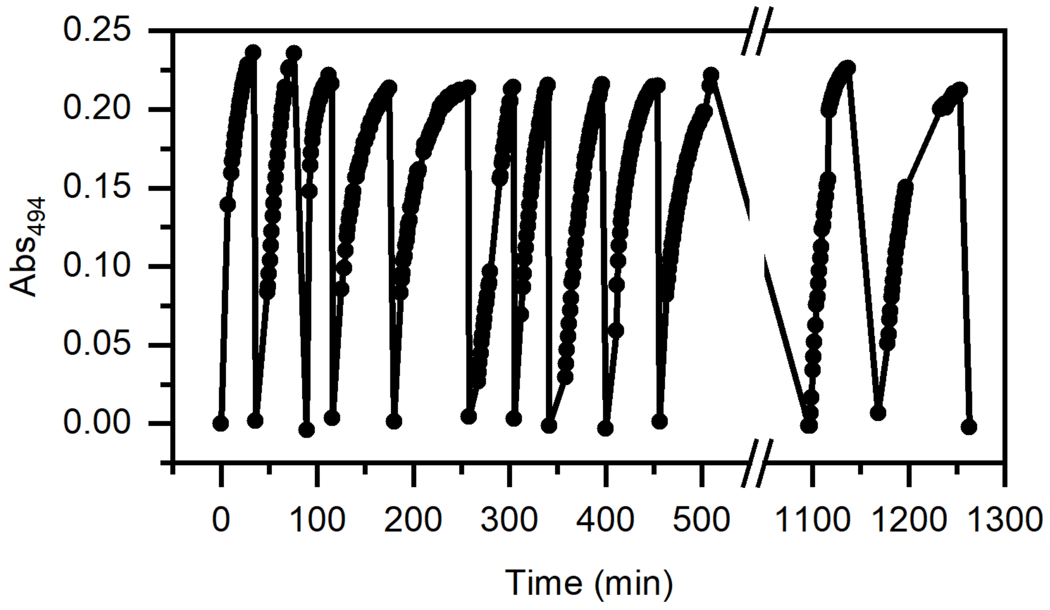

Supplementary Figure 8. FeMETPsc1 redox cycling experiment time course. Spectra were generally acquired every minute except when air was purged in the solution right after dithionite addition. Time axis break corresponds with the overnight reduction under Ar .

2.3 ZnMC6*a as photosensitizer in a photo-triggered electron cascade

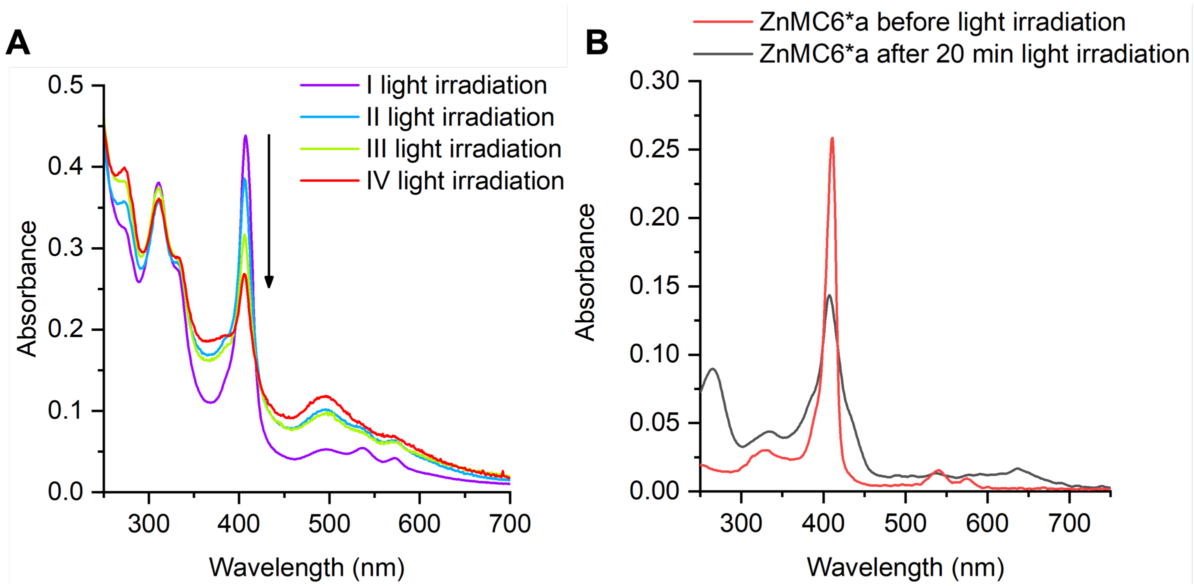

Supplementary Figure 9. A) UV-vis spectra acquired after each cycle of light irradiation in the electron transport experiment with FeMETPsc1. The arrow indicates the variation of the Soret band absorbance over subsequent cycles, showing increasing degradation of ZnMC6*a. **B)** UV-Vis spectra of ZnMC6*a acquired before (red line) and after (black line) exposure to 20 min of light irradiation in the absence of FeMETPsc1.

2.4. Synthesis and purification

METPsc1 was chemically synthesized on a 0.1 mmol scale by the standard Fmoc-protocols of solid-phase peptide synthesis, obtaining the isolated crude product in 65% yield, based on the resin substitution level. The RP-HPLC chromatogram (Supplementary Fig. 10) shows the presence of a main peak at 36.597 min (about 50% purity), corresponding to the desired product. Its identity was ascertained by ESI MS analysis, which gave an experimental mass of (2968 ± 1) Da, in agreement with the theoretical mass value (2967.25 Da).

The crude peptide was purified to homogeneity by preparative RP-HPLC with a final overall yield of the isolated pure product of 30%, and its purity (>99%) and identity was assessed by analytical RP-HPLC and MS analyses (Supplementary Fig. 11 and 12).

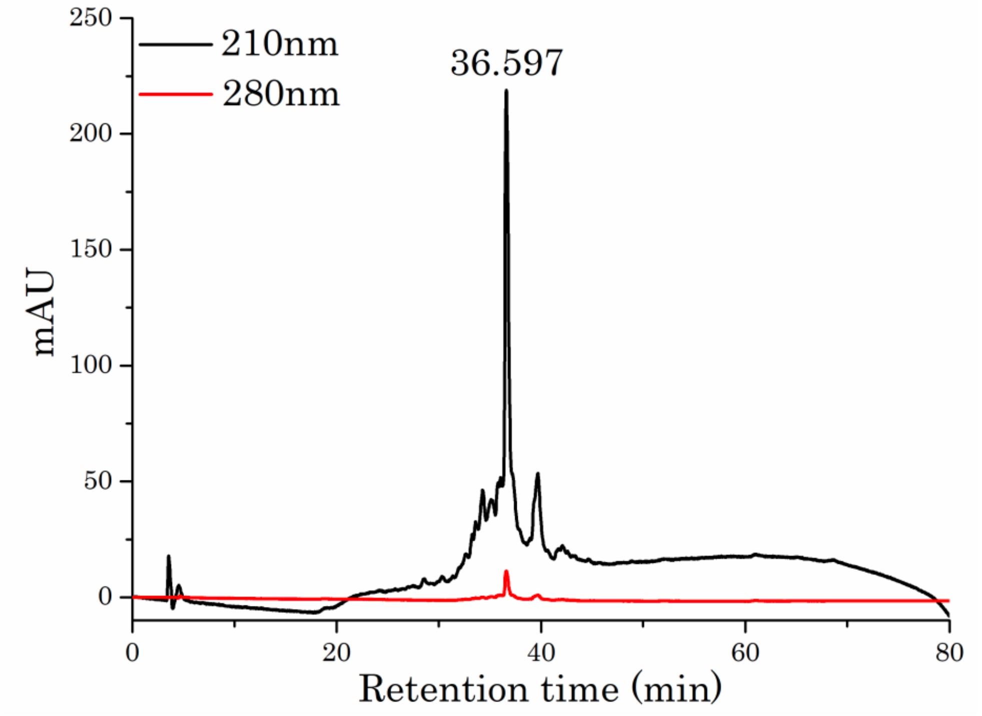

Supplementary Figure 10. RP-HPLC chromatogram from the LC-MS analysis of crude METPsc1.

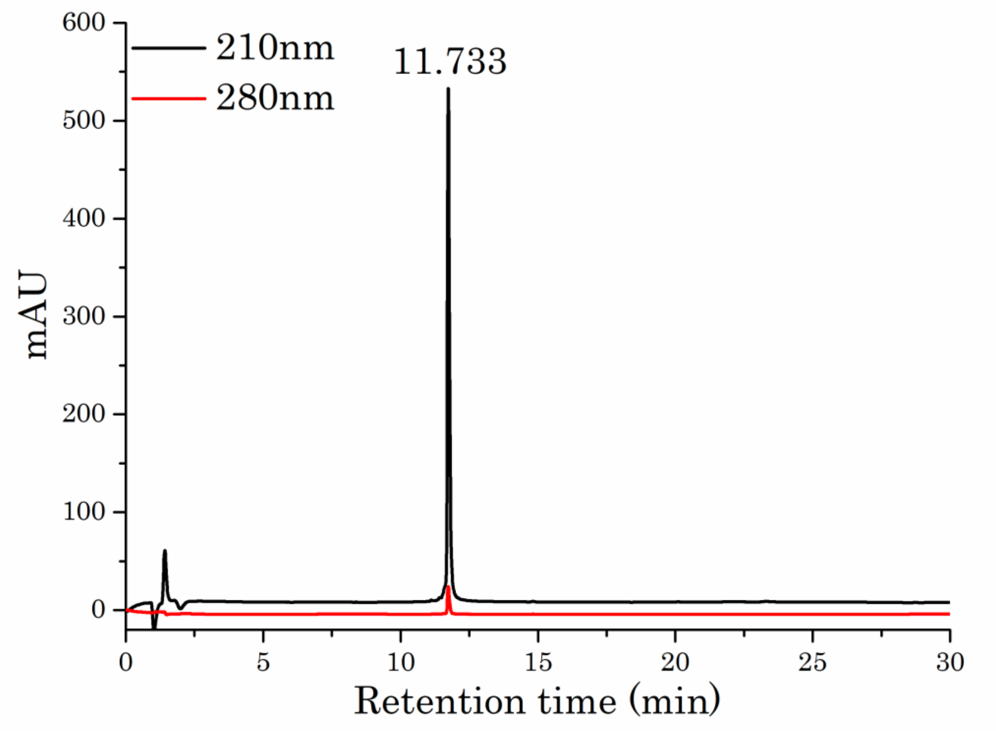

Supplementary Figure 11. RP-HPLC analytical chromatogram of pure METPsc1.

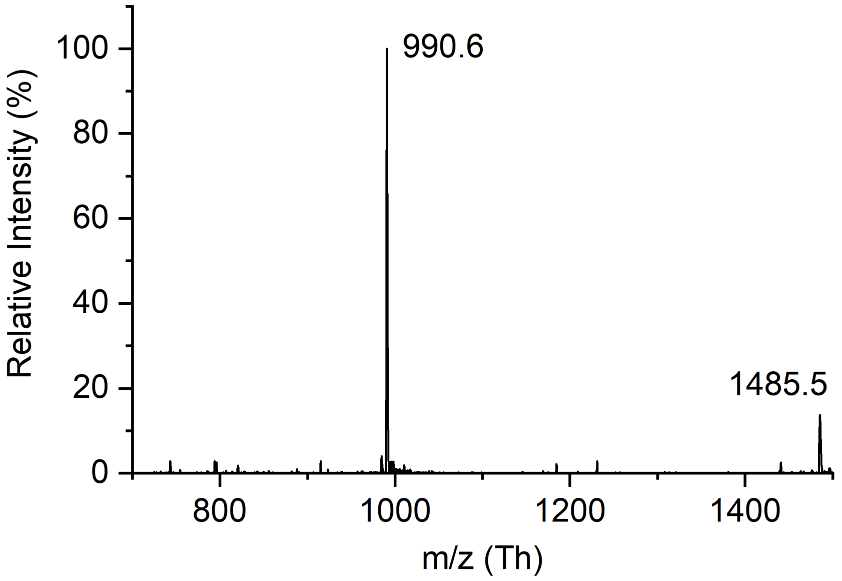

Supplementary Figure 12: ESI-MS spectrum of METPsc1. The signal at m/z = 1485.5 Th corresponds to the $[M+2H^{+}]^{2+}$ ion (theoretic isotopic mass: 1485.1 Da); the signal at m/z = 990.6 Th corresponds to the $[M+3H^{+}]^{3+}$ ion (theoretic isotopic mass: 990.4 Da).

3. Supplementary References

[1] L. L. C. Schrödinger, **2010**.

[2] J. Zhou, G. Grigoryan, *Protein Sci.* **2015**, *24*, 508–524.

[3] G. E. Crooks, G. Hon, J.-M. Chandonia, S. E. Brenner, *Genome Res.* **2004**, *14*, 1188–1190.

[4] S. J. Fleishman, A. Leaver-Fay, J. E. Corn, E.-M. Strauch, S. D. Khare, N. Koga, J. Ashworth, P. Murphy, F. Richter, G. Lemmon, J. Meiler, D. Baker, *PLOS ONE* **2011**, *6*, e20161.

[5] H. Park, P. Bradley, P. Greisen, Y. Liu, V. K. Mulligan, D. E. Kim, D. Baker, F. DiMaio, *J. Chem. Theory Comput.* **2016**, *12*, 6201–6212.

[6] N. F. Polizzi, Y. Wu, T. Lemmin, A. M. Maxwell, S.-Q. Zhang, J. Rawson, D. N. Beratan, M. J. Therien, W. F. DeGrado, *Nat. Chem.* **2017**, *9*, 1157–1164.

[7] S. La Gatta, L. Leone, O. Maglio, M. De Fenza, F. Nastri, V. Pavone, M. Chino, A. Lombardi, *Molecules* **2021**, *26*, 5221.

[8] C. A. Smith, T. Kortemme, *J. Mol. Biol.* **2008**, *380*, 742–756.

[9] L. G. Nivón, R. Moretti, D. Baker, *PLOS ONE* **2013**, *8*, e59004.

[10] V. Pavone, *Int. J. Biol. Macromol.* **1988**, *10*, 238–240.

[11] V. Pavone, G. Gaeta, A. Lombardi, F. Nastri, O. Maglio, C. Isernia, M. Saviano, *Biopolymers* **1996**, *38*, 705–721.

[12] B. Dasgupta, P. Chakrabarti, *BMC Struct. Biol.* **2008**, *8*, 39.

[13] P. Y. Chou, G. D. Fasman, *J. Mol. Biol.* **1977**, *115*, 135–175.
